# Antisite defect qubits in monolayer transition metal dichalcogenides

**DOI:** 10.1038/s41467-022-28133-x

**Published:** 2022-01-25

**Authors:** Jeng-Yuan Tsai, Jinbo Pan, Hsin Lin, Arun Bansil, Qimin Yan

**Affiliations:** 1 Department of Physics, Temple University, Philadelphia, PA 19122 USA; 2 Institute of Physics, Academia Sinica, Taipei, Taiwan; 3 Physics Department, Northeastern University, Boston, MA 02115 USA

**Keywords:** Qubits, Two-dimensional materials

## Abstract

Being atomically thin and amenable to external controls, two-dimensional (2D) materials offer a new paradigm for the realization of patterned qubit fabrication and operation at room temperature for quantum information sciences applications. Here we show that the antisite defect in 2D transition metal dichalcogenides (TMDs) can provide a controllable solid-state spin qubit system. Using high-throughput atomistic simulations, we identify several neutral antisite defects in TMDs that lie deep in the bulk band gap and host a paramagnetic triplet ground state. Our in-depth analysis reveals the presence of optical transitions and triplet-singlet intersystem crossing processes for fingerprinting these defect qubits. As an illustrative example, we discuss the initialization and readout principles of an antisite qubit in WS_2_, which is expected to be stable against interlayer interactions in a multilayer structure for qubit isolation and protection in future qubit-based devices. Our study opens a new pathway for creating scalable, room-temperature spin qubits in 2D TMDs.

## Abstract (web summary)

Two-dimensional materials offer a promising platform for scalable solid-state spin qubit. Here, using high-throughput ab initio simulations, the authors identify suitable defect centers in monolayer group-VI transition metal dichalcogenides and assess their potential as qubits.

## Introduction

The ongoing second quantum revolution calls for exploiting the laws of quantum mechanics in transformative new technologies for computation and quantum information science (QIS) applications^1^. Spin qubits based on solid-state defects have emerged as promising candidates because these qubits can be initialized, selectively controlled, and readout with high fidelity at ambient temperatures^2,3^. Solid-state defects, especially in 2D TMDs offer advantages of scalability and ease of device fabrication. Point defects as spin qubits have been demonstrated in traditional semiconductor systems^4^, including the nitrogen-vacancy (NV^−^) center in diamond and the spin-1/2 defect in doped silicon^3,5–8^ among other possibilities^4,9–11^. In particular, the Si-vacancy complex in diamond^11^, vacancy defects in SiC^12^, and vacancy complexes in AlN^10^ have been predicted as qubits. A neutral divacancy (*V*_C_-*V*_Si_)^0^ in SiC has been identified as a qubit with millisecond coherence time^13^, where an improvement in dephasing time by over four orders of magnitude can be achieved by embedding the qubit in a decoherence-protected subspace through microwave dressing^14^.

A key challenge in the development of controllable multiple-qubit systems is how to effectively couple spin defects and achieve high fidelity and long coherence times. The planar structures of atomically thin 2D materials present a superior platform for realizing controlled creation and manipulation of defect qubits with better potential for scalability than the bulk materials. In 2D materials, defects can be generated by a number of existing approaches^15^, and characterized and manipulated using atomic-level scanning probe techniques^16^. The carbon-vacancy complex (C_B_-*V*_N_) in hexagonal boron nitride (h-BN) has emerged as the first such qubit^17^. Nitrogen-vacancy complex defect (N_B_-*V*_N_) and the negatively charged boron vacancy defect (*V*_B_) have also been proposed as qubit candidates in h-BN^18,19^, and a number of point defects in h-BN show promise as ultrabright single-photon emitters at room temperature^20,21^.

TMDs are a major class of 2D graphene cognates that are attracting intense current interest because of their sizable band gaps and high absorption coefficients, among other unique physical and chemical properties. Atomic defects in as-grown TMD samples, such as the anion vacancies^22^, are well known to play an essential role in their electronic behavior^23^. Compared to 3D wide-bandgap materials, the spin coherence time in MoS_2_ has been estimated to be extremely long, on the order of 30 ms, suggesting the potential of TMDs as good host materials for multiple-qubit operation^24^. The field is thus ripe for the discovery and rational design of promising defect qubits in 2D materials and their implementation in single- and multi-qubit platforms for QIS applications.

Here, we report the identification of anion-antisite defects M_X_ in six MX_2_ (M: Mo, W; X: S, Se, Te) TMD systems as promising 2D solid-state defect qubits obtained via a high-throughput search based on a new qubit formation hypothesis involving symmetry constraints as well as the host electronic structures. Our first-principles defect computations, see the Methods section below for details, demonstrate that the proposed antisites in these TMDs host paramagnetic triplet ground states with flexible level splittings controlled by site symmetries and both the in-plane and out-of-plane *d* orbital interactions. Taking W_S_ antisite in WS_2_ as an especially favorable case, we demonstrate a viable transition loop between the triplet and singlet defect states, including optical excitations/relaxations and nonradiative decay paths for the W_S_ antisite as a qubit. A complete set of qubit operational processes, including initialization, manipulation, and readout steps is delineated to provide a blueprint for experimental verification.

## Results

### Qubit discovery hypothesis

Our data-driven defect qubit discovery effort in the TMDs is based on satisfying three major descriptors as follows. (1) A paramagnetic, long-lived triplet ground state with a stable charge state and multiple in-gap defect levels. (2) An optical transition path between the ground and excited triplet states, as well as a spin-selective (nonradiative) decay path between the different spin-multiplet states for qubit initialization. And, (3) distinguishable luminescence signatures for the two spin sublevels for qubit readout^4^.

Before we turn to discuss how the interplay of the host electronic structure and local site-symmetry yields an anion antisite in the TMDs as a viable defect qubit, we note that wide bandgap compounds, such as SiC, AlN, and h-BN, are mostly characterized by occupied anion states as valence bands and unoccupied cation states as conduction bands. As a result, cation (anion) vacancy defect levels that originate from anion (cation) dangling-bond states are usually located in the valence (conduction) band. Therefore, it becomes necessary to introduce impurities next to the vacancies^4^ or apply strain perturbations^10^ in the wide bandgap systems to create additional energy splittings to push the defect levels into the gap. Monolayer group-VI TMDs possess fundamentally different electronic structures that are characterized by dominant *d*-states of the transition metals contributing to the valence and conduction band edges^25^. As a result, point defects created by cations such as the anion antisites and anion vacancies/complexes are more likely to host deep in-gap defect levels. Notably, intrinsic defects including vacancies and anion antisites have been observed experimentally in the TMDs^26^.

It is useful to recall here that a triplet ground state is preferred (Hund’s rule) when the exchange energy involving the interaction of two parallel spins is favorable compared to the energy required to lift one of the electrons to a higher level. In other words, either a small or zero energy splitting between the two highest occupied levels is a prerequisite for stabilizing the triplet ground state. An energetically favorable scenario is that the local site-symmetry of the point defect belongs to a point group with at least one 2-dimensional (2D) irreducible representation (IR). An operational definition of IR is that the direct sum of IRs can decompose a completely reducible representation. In this way, the IRs of the point group can decompose the finite-dimensional space formed by the defect levels and label these levels in the bandgap, allowing the selection rules to be determined. The 2D IR may generate doubly-degenerate defect levels and hence a strong tendency to create a triplet ground state when these two levels are the highest occupied levels as is the case for the NV center in diamond. *d* states of transition metal cations in TMDs tend to have relatively large exchange energies, which favor a triplet ground state in keeping with the Hund’s rule.

### Antisites in group-VI TMDs: a promising qubit platform

Based on the preceding discussion of our hypothesis, we performed a symmetry-based data-mining search to identify nonmagnetic and relatively stable MX_2_ TMD compounds from the 2D materials database C2DB^27^ with computed band gaps larger than 1.4 eV and energies above the convex hull less than 0.1 eV/atom. 27 TMD compounds were identified and assigned to three different phases, 1H, 1 T, and 1 T’ and the corresponding point groups, *D*_*3h*_, *D*_*3d*_, and *C*_*2h*_, respectively. 1 T’ phase is ruled out since the *C*_*2h*_ point group does not have a 2D IR. For group-VI TMDs, the 1H phase is more stable than the 1 T phase under equilibrium conditions^28^. Therefore, we focused on six nonmagnetic group-VI 1H TMDs MX_2_ (M: Mo, W; X: S, Se, Te). We performed high-throughput defect computations and found that no neutral anion vacancy in these six group-VI TMDs hosts a triplet ground state, which is largely due to the fact that the cation dangling-bond states surrounding anion vacancies form a state space consisting of fully occupied 1D *a*_*1*_ and unoccupied 2D *e* levels. As a result, the anion vacancies do not favor a triplet spin configuration. Note that anion vacancies with a 2- charge state host triplet ground states, but the lack of unoccupied in-gap defect levels makes the necessary excitation process impossible, see Supplementary Note 1 for details. We thus ruled out isolated anion vacancies and focused on anion-antisite defects in the 1H TMDs.

**Fig. 1:**
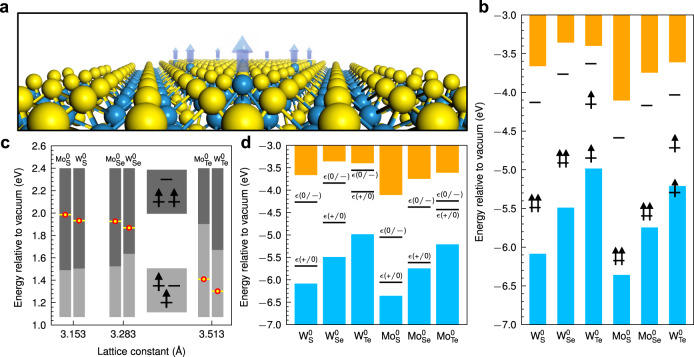
Anion-antisite defects in six 1H transition metal dichalcogenides. **a** A schematic illustration of the M_X_^0^ antisite defect in monolayer 1H-TMD. **b** In-gap defect levels of M_X_^0^ in six 1H-TMDs with triplet ground states. Blue and orange colored bars represent the valence and conduction bands of the host materials. Note that M_S/Se_^0^ has doubly-degenerate highest-occupied defect levels, whereas in the case of M_Te_^0^ there is a splitting between the occupied defect levels. **c** Correlation between the defect-level splittings and the *z*-positions of the antisites relative to those of the neighboring cations. Yellow dots (red-enclosed) mark the equilibrium *z*-positions of the antisite defects. The *z*-positions corresponding to the two level-splitting patterns of the defects, as illustrated in the two insets (upper: 2–1 type splitting; lower: 1–2 type splitting), are shown by dark-grey and grey bars, respectively. Note that a further Jahn-Teller splitting of the doubly-degenerate levels occurs in the 1/2 splitting case. **d** Thermodynamic transition levels for the six antisite defects in 1H-TMDs. *ϵ*(+/0) and *ϵ*(0/-) denote the transition levels from the charge state 1+ to 0, and from 0 to 1-, respectively. Neutral charge states are thermodynamically stable when the Fermi level is close to the mid-gap.

Figure 1a presents an example of an anion antisite in TMDs where a cation is located on an anion site in the crystal lattice. The location and occupation of defect levels created by the antisite are controlled by its interaction with the three cation atoms in the central atomic layer as well as the defect charge state (see Methods). Defect levels of six anion antisites M_X_^0^ in the band gaps of 1H-MX_2_ (M: Mo, W; X: S, Se, Te) were computed and those of the neutral antisites are shown in Fig. 1b. It is remarkable that all six 1H TMDs host neutral antisites in a triplet ground state. Note that it has been predicted that anion antisite in WS_2_^29^ and MoS_2_^30^ favors a triplet state. In spite of the universal presence of a triplet ground state in these six antisite systems, we emphasize that the level splittings for the three defect levels in the spin-up channel are not uniform (Fig. 1b). Constructed mainly from $d_{{{\mathrm{x}}}^{2}{\mbox{-}}{{{\mathrm{y}}}^{2}}}$, *d*_xy_, and $d_{{{\mathrm{z}}}^{2}}$ orbitals of the cation atoms located at the antisites, the three defect levels of the six 1H TMDs fall into two level-splitting patterns characterized by the position of the $d_{{{\mathrm{z}}}^{2}}$ level relative to the $d_{{{\mathrm{x}}}^{2}{\mbox{-}}{{{\mathrm{y}}}^{2}}}$ and *d*_xy_ levels. For the neutral antisites in MoS_2_, MoSe_2_, WS_2_, and WSe_2_, the two highest occupied levels in the gap are doubly degenerate, while those in MoTe_2_ and WTe_2_ generate three discrete defect levels in the band gaps of the host material.

In order to gain insight into the differences in defect-level splittings in various 1H TMDs, we adopt a local-symmetry analysis. The local symmetry for the unperturbed environment of an antisite is *C*_*3v*_, which is a subgroup of the crystal point-group *D*_*3h*_ of the pristine 1H-MX_2_ systems. In 1H-MX_2_ compounds, the *d* orbitals hybridize and transform into 2D IR *E* and 1D IR ${A}_{1}$. Within this local symmetry, $d_{{{\mathrm{x}}}^{2}{\mbox{-}}{{{\mathrm{y}}}^{2}}}$ and *d*_xy_ orbitals at the antisites belong to the 2D IR *E* while the $d_{{{\mathrm{z}}}^{2}}$ orbital belongs to the 1D IR ${A}_{1}$. The doubly degenerate $d_{{{\mathrm{x}}}^{2}{\mbox{-}}{{{\mathrm{y}}}^{2}}}$ and *d*_xy_ orbitals at the ansitites interact with the three neighboring cations mainly in the *x*-*y* plane. Their energy levels are therefore affected by the M-M distances. On the other hand, the interaction between the $d_{{{\mathrm{z}}}^{2}}$ orbitals of the antisites and the three cations is determined by the location of the antisite defect along the *z*-direction relative to the cation layer.

Our analysis indicates that, due to variations in the overlap of the wavefunctions of the antisite with the adjacent cations, the $d_{{{\mathrm{z}}}^{2}}$ defect level shifts up in energy relative to the $d_{{{\mathrm{x}}}^{2}{\mbox{-}}{{{\mathrm{y}}}^{2}}}$ and *d*_xy_ levels as the antisite moves away from the cation layer along the *z*-direction (see Supplementary Note 10 for details). This relative energy shift eventually reaches a critical point where a level-switching takes place. As shown in Fig. 1c, the position of the antisite along the *z*-direction in equilibrium structure (red-enclosed yellow dot in Fig. 1c) is negatively correlated with the lattice constant. This is mainly due to the difference in atomic sizes between the antisite and the anions, so that the equilibrium *z*-positions of the antisites in S- and Se-based TMDs are higher than those in Te-based TMDs, see Supplementary Note 11 for details. The equilibrium *z*-positions of the antisites thus depend on the anion species or lattice constants, and lead to a clear correlation between the level-splitting patterns and the anion species involved. For antisites in MoS_2_, MoSe_2_, WS_2_, and WSe_2_, the $d_{{{\mathrm{x}}}^{2}{\mbox{-}}{{{\mathrm{y}}}^{2}}}$ and *d*_xy_ levels are located below the $d_{{{\mathrm{z}}}^{2}}$ level (2/1 splitting), while the opposite is the case for MoTe_2_ and WTe_2_ (1/2 splitting).

Due to interactions between the antisite and adjacent cation atoms, the neutral antisite has two extra electrons after bonding, which can occupy two defect levels in the gap, creating two occupied levels and one unoccupied level in the up-spin channel in the triplet state. In the case of 2/1 splitting, the lowest two $d_{{{\mathrm{x}}}^{2}{\mbox{-}}{{{\mathrm{y}}}^{2}}}$ and *d*_xy_ levels are occupied and remain doubly degenerate. In the case of 1/2 splitting (MoTe_2_ and WTe_2_), in contrast, a single electron occupying $d_{{{\mathrm{x}}}^{2}{\mbox{-}}{{{\mathrm{y}}}^{2}}}$ and *d*_xy_ levels introduces a sizable spontaneous level splitting due to the magnetic Jahn-Teller effect^31^, which stabilizes the defect system by lowering the symmetry of the magnetization density without the distortion of ionic configuration. A small perturbation from this metastable configuration may introduce a Jahn-Teller distortion that reduces the local-symmetry *C*_*3v*_ approximately to the local-symmetry *C*_*h*_. We emphasize that different level-splitting patterns of neutral antisites in the 1H-TMD family originate from the unique anisotropic orbital interactions in 2D materials.

In order to evaluate the stabilities of neutral antisite defects in the six 1H TMDs, we have calculated the thermodynamic charge-transition levels (i.e. the Fermi-level positions at which the most stable charge state of a defect changes, see Methods) shown in Fig. 1d. Charge-state corrections for the charged antisite systems were obtained by using an extrapolation method, see Methods^32,33^. Energy windows for the Fermi level in the gap where the neutral charge state is most stable are: 1.43 eV, 0.88 eV, 0.48 eV, 1.01 eV, 1.24 eV, 0.19 eV for W_S_^0^, W_Se_^0^, W_Te_^0^, Mo_S_^0^, Mo_Se_^0^, and Mo_Te_^0^, respectively (Supplementary Fig. 2). The thermodynamic transition levels, obtained via the hybrid functional calculations, are expected only to capture the trends in defect stabilities. However, it is reasonable to expect that neutral antisite defects will be stable in these 1H-TMDs when the Fermi level is close to the mid-gap. Note that although the as-grown monolayer MoS_2_ is usually *n*-type, there is strong experimental evidence that the Fermi energy in monolayer TMDs can be controlled effectively^34,35^. Furthermore, the Fermi level in ultrathin 2D systems can be tuned via electrostatic gating^36^. The computed formation energies for the neutral antisites are relatively high, indicating that the intrinsic concentration of antisite defects in as-grown TMDs under equilibrium conditions would be relatively low, see Supplementary Note 9 for details. Note that the anion-antisite defects have been observed experimentally in both WS_2_ and WSe_2_^26,37^, and that these defects could be created under nonequilibrium conditions using irradiation or ion implantation.

For a viable defect qubit, the defect levels related to qubit operation must be deep in the gap to minimize effects of disruptive interactions with the bulk states. The highest occupied defect levels of Mo_s_^0^, Mo_Se_^0^, Mo_Te_^0^, and W_Te_^0^ lie close (within 0.3 eV) to the valence band maximum (VBM) of the host materials. In contrast, the defect levels in W_S_^0^ and W_Se_^0^ are sufficiently deep (about 0.6 eV above the VBM) for qubit operation^38^. Among the six anion antisites, W_S_^0^ and W_Se_^0^ are therefore the most promising candidates as defect qubits in 2D TMDs.

### Antisite defect qubit in WS_2_

**Fig. 2:**
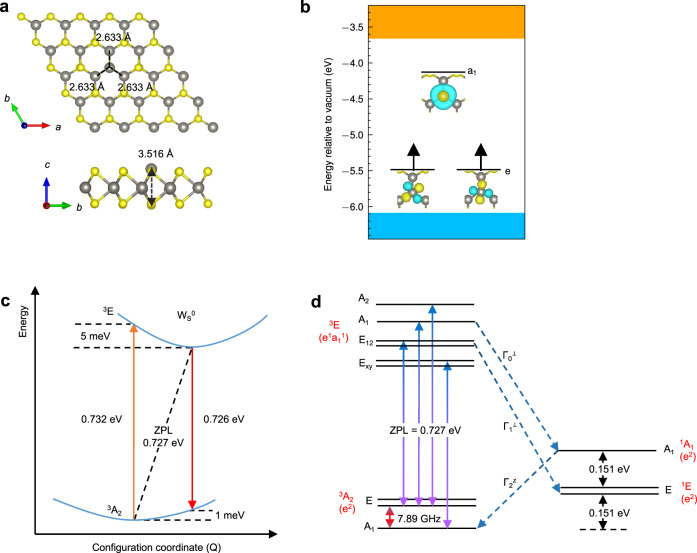
Electronic and geometric structure of the neutral antisite defect W_S_^0^ in WS_2_. **a** Optimized structure of the antisite defect W_S_^0^ in WS_2_, showing its local-symmetry *C*_*3v*_. **b** Energy diagram showing the defect levels in the triplet ground state ^3^*A*_*2*_. The defect levels *e* and *a*_*1*_ are mainly composed of {$d_{{{\mathrm{x}}}^{2}{\mbox{-}}{{{\mathrm{y}}}^{2}}}$, *d*_xy_} and $d_{{{\mathrm{z}}}^{2}}$ orbitals of the defect. The depictions of the wavefunctions involved are shown. **c** Configuration coordinate diagram of W_S_^0^ in WS_2_ for the triplet ground state ^3^*A*_*2*_ and the triplet excited state ^3^*E* with inclusion of spin-orbit coupling. **d** Sublevels for the triplet ground state ^3^*A*_*2*_, the triplet excited state ^3^*E*, and the singlet states ^1^*E* and ^1^*A*_*1*_, labeled by the IRs of *C*_*3v*_. Spin-conserving optical transitions are shown by colored solid arrows. Symmetry-allowed intersystem-crossing paths are denoted by dashed arrows. The labels {*Γ*_0_^⊥^, *Γ*_1_^⊥^} and *Γ*_2_^z^ indicate the allowed intersystem-crossing paths via the nonaxial spin-orbit coupling and the axial spin-orbit coupling, respectively. The energy difference between ^3^*A*_*2*_ and ^1^*E* was calculated with inclusion of spin-orbit coupling.

We now discuss W_S_^0^ in WS_2_ as a benchmark system to demonstrate the operation principle of antisite defect qubits. A pristine monolayer of WS_2_ in the H-phase (Supplementary Table 2) is composed of three hexagonal layers that form a sandwich-like structure (S-W-S). We define the direction of the *c* lattice vector as the *z*-axis. Sulfur atoms occupy the upper and lower hexagonal sublattice sheets with a symmetrical W plane lying between these sheets. Our optimized structure indicates that the W-S and S-S distances are 2.391 Å and 3.107 Å, respectively^28^. Hybrid functional calculations predict a bandgap of 2.42 eV which is close to the experimental value of about 2.41 eV^39^. The optimized structure of the anion-antisite W_S_^0^ is shown in Fig. 2a. The local environment of W_S_^0^ without perturbation has symmetry *C*_*3v*_ with the rotation axis lying along the *z*-direction. Note that if the antisite in WS_2_ is initially perturbed by a random displacement, the symmetry of the resulting structure can be lowered from *C*_*3v*_ to *C*_*h*_ with lower energy of ~25 meV per unit cell compared to the metastable structure with symmetry *C*_*3v*_, see Supplementary Note 6 for details. The extent of the symmetry-driven lowering can be estimated by calculating the zero-field splitting (ZFS) of the sublevels *S*_*x*_ (*m*_*s*_ = 1) and *S*_*y*_ (*m*_*s*_ = −1) due to the spin-spin dipolar interactions. The analysis shows that the ratio of the splitting between *m*_*s*_ = 0 and *m*_*s*_ = ±1 and the splitting between *m*_*s*_ = 1 and *m*_*s*_ = −1 is about 5:1, implying that the deviation from *C*_*3v*_ is relatively small. As a result, the qubit operation principle in the case of *C*_*h*_ resembles that for *C*_*3v*_, see Supplementary Notes 6 and 7 for details.

The calculated electronic structure of W_S_^0^ hosts a triplet ground state. The in-gap defect levels can be labeled by IRs of the point-group *C*_*3v*_ as shown in Fig. 2b. The ground and excited states of W_S_^0^ are described by single Slater determinants as *e*^2^ and *a*_*1*_^1^*e*^1^, respectively. Note that one can equivalently express a many-body state by either the electron or hole occupation of the single-particle orbitals^40^. From this point of view, the defect levels of ${{{{{\rm{N}}}}}}{{{{{{\rm{V}}}}}}}^{-}$ center in diamond (hole occupation) are identical to the defect levels of W_S_^0^ (electron occupation) in terms of single Slater determinants^40,41^. Therefore, we will adopt the state symbols for *e*^2^ and *a*_*1*_^1^*e*^1^ as {^3^*A*_*2*_, ^1^*E*, ^1^*A*_*1*_} and ^3^*E*, respectively. The defect levels calculated with the inclusion of spin-orbit interactions are shown in Supplementary Note 12.

In order to access transition processes involving the triplet ground state ^3^*A*_*2*_ and the triplet excited state ^3^*E*, we performed constrained DFT (CDFT) calculations in which occupations of the Kohn-Sham orbitals are constrained to desirable configurations. We set the occupation of defect levels as *e*^0.5^*e*^0.5^*a*_*1*_^1^ for the excited state. This methodology combined with HSE calculations has been applied to evaluate the zero-phonon line (ZPL) of ${{{{{\rm{N}}}}}}{{{{{{\rm{V}}}}}}}^{-}$ center to achieve excellent agreement with experiments^4,42^. Other possible occupation configurations for the excited states were considered as well and the corresponding transition processes are presented in Supplementary Note 13. As shown in the configuration coordinate diagram (Fig. 2c), which includes the consideration of spin-orbit interactions (see Supplementary Note 5 for details), the ZPL for the internal transition between the triplet ground and excited states is 0.727 eV (corresponding wavelength of ~1.7 µm), which lies in the near-infrared (NIR) range. Frank-Condon relaxation energies are 5 meV and 1 meV for the excited and ground states, respectively. These extremely small vibrational couplings in the internal transitions imply that antisite defects in TMDs will likely be suitable for other QIS applications such as single-photon emitters and quantum sensors.

Positions of singlet states are significant for nonradiative decay paths that connect triplet and singlet states. We estimate positions of singlet states ^1^*E* and ^1^*A*_*1*_ by considering the Coulomb interaction^41,43^. Note that since the singlet state ^1^*A*_*1*_ is a strongly correlated state, it cannot be described accurately as a single-particle Kohn-Sham state. Since we have the same local symmetry, we can adopt the results from the ${{{{{\rm{N}}}}}}{{{{{{\rm{V}}}}}}}^{-}$center^41^ based on group theory in which the ratio of energy shifts for ^1^*E* and ^1^*A*_*1*_ relative to ^3^*A*_*2*_ is 1:2. The energy difference obtained by first-principles calculations between ^1^*E* and ^3^*A*_*2*_ is 0.151 eV, from which the energy difference between ^1^*A*_*1*_ and ^3^*A*_*2*_ can be estimated to be 0.302 eV. Considering that the ZPL of the triplet states is 0.727 eV, it indicates that the singlet states ^1^*E* and ^1^*A*_*1*_ are located between the triplet excited state ^3^*E* and the triplet ground state ^3^*A*_*2*_ (Fig. 2d). Note that the energy difference between ^3^*A*_*2*_ and ^1^*E* here is calculated including the effects of the spin-orbit coupling.

In order to operate as a qubit, a defect center must have distinct signatures of optical transitions involving various sublevels and support nonradiative decay paths^12^. The spin-orbit coupling (SOC) effects and the associated sublevels are important for ascertaining the allowed intersystem crossings (ISCs) between different spin configurations^44^. We analyzed the matrix element of the single-particle spin-orbit operator ${\hat{H}}_{{SO}}$within a group-theory framework to determine the symmetry-allowed intersystem crossings, see Supplementary Note 8 for details. Three allowed intersystem-crossing paths *Γ*_0_^⊥^, *Γ*_1_^⊥^, and *Γ*_2_^z^ are identified based on spin quantum numbers and IRs of tensor products of wavefunctions and spinors. The allowed spin-conserving optical transitions and intersystem crossings are shown in Fig. 2d. *Γ*_0_^⊥^ and *Γ*_1_^⊥^ involve nonaxial components of ${\hat{H}}_{{SO}}$, while *Γ*_2_^z^ involves the axial component. Beyond W_S_^0^ in WS_2_, detailed properties of antisite qubit W_Se_^0^ in WSe_2_ are discussed in Supplementary Note 4. For antisite defects Mo_S_^0^ in MoS_2_ and Mo_Se_^0^ in MoSe_2_, computed properties including the ZPLs and ZFSs in the ground states are presented in Supplementary Note 5.

### Qubit operation principle

**Fig. 3:**
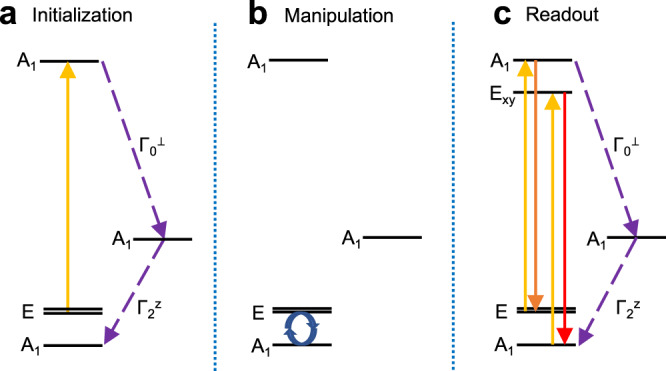
Operational loop for the antisite qubit W_S_^0^. **a** Initialization: The defect center is pumped optically (gold line) from the sublevel *E* in the triplet ground state ^3^*A*_*2*_ to the sublevel *A*_*1*_ in the triplet excited state ^3^*E*, and then the defect center relaxes back to the sublevel *A*_*1*_ in the triplet ground state via the intersystem-crossing paths *Γ*_0_^⊥^ and *Γ*_2_^z^ (dashed purple lines). **b** Manipulation: The qubit can be manipulated by using electron paramagnetic resonance (EPR) on one of the sublevels *E* and the sublevel *A*_*1*_ in the triplet ground state. The blue circular arrows indicate the manipulation process via a microwave pulse. **c** Readout: The defect center is optically pumped again, and the intensities of fluorescence involving different initial states are detected. Note that the fluorescence process *E*_*xy*_(^3^*E*) → *A*_*1*_(^3^*A*_*2*_) (red line) has a higher intensity than the process *A*_*1*_(^3^*E*) → *E*(^3^*A*_*2*_) (orange line) due to the existence of intersystem-crossing transitions (dashed purple lines) that weakens the intensity of the radiative transition.

A complete loop for qubit operation based on W_S_^0^ in WS_2_ is illustrated in Fig. 3. The initialization, manipulation, and readout of our TMD-based antisite qubit resemble the operations of defect qubits in the well-known ${{{{{\rm{N}}}}}}{{{{{{\rm{V}}}}}}}^{-}$ center in diamond^4,12,45^. We choose the sublevel *A*_*1*_ (*m*_*S*_ = 0) and one of sublevels in *E* (*m*_*s*_ = ±1) in the triplet ground state ^3^*A*_*2*_ as a two level qubit system. The energy gap between the sublevels *A*_*1*_ and *E*, which is dominated by the spin-spin dipolar coupling, is an important parameter for qubit operation that bears on the spin-lattice relaxation time *T*_*1*_^46^. We calculated ZFS tensor **D** based on pseudo-wavefunctions (Supplementary Note 5), which have yielded reasonable results for the ${{{{{\rm{N}}}}}}{{{{{{\rm{V}}}}}}}^{-}$ center^45,47^. The computed ZFS of 7.89 GHz is more than twice as large as that in ${{{{{\rm{N}}}}}}{{{{{{\rm{V}}}}}}}^{-}$ center (2.88 GHz)^7^. The ZFSs for anion antisites M_X_^0^ in MX_2_ (M: Mo, W; X: S, Se) are shown in Supplementary Table 5.

Initialization of the qubit could be achieved by optically pumping the defect center from the sublevel *E* in the triplet ground state ^3^*A*_*2*_ to the sublevel *A*_*1*_ in the triplet excited state ^3^*E* (Fig. 3a). The sublevel *A*_*1*_ in the triplet excited state has an allowed intersystem crossing to the sublevel *A*_*1*_ in the singlet state ^1^*A*_*1*_ via path *Γ*_0_^⊥^, and then the system relaxes back to the sublevel *A*_*1*_ in the triplet ground state via path *Γ*_2_^z^. The preceding transition processes form a complete cycle for the initialization of the qubit. Manipulation of the qubit could be implemented by utilizing one of the sublevels *E* and the sublevel *A*_*1*_ in the triplet ground state by applying resonant microwave (Fig. 3b). The fluorescence intensity from sublevels *E* is expected to be weaker than that from the sublevel *A*_*1*_ due to the existence of the intersystem-crossing path *Γ*_0_^⊥^. Readout of the qubit can therefore be realized by detecting the difference in fluorescence intensity involving different qubit states (Fig. 3c). The set of operations presented above would enable qubit initialization, manipulation, and readout, forming the essential operation principles for antisite qubits in TMDs.

### Qubit protection scheme and spin coherence

**Fig. 4:**
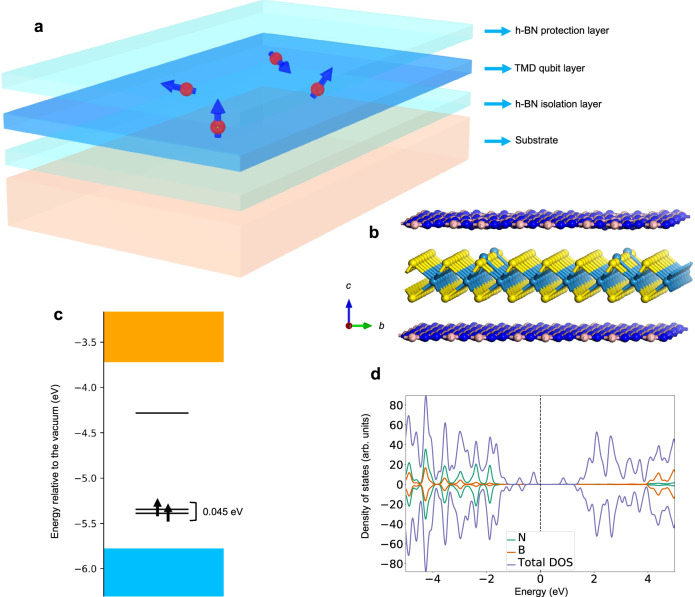
Illustration of an environmentally protected qubit design based on the h-BN/WS_2_/h-BN heterojunction structure. **a** Schematic of the proposed 2D-heterojunction structure. **b** Schematic of the optimized heterojunction consisting of 2$\sqrt{7}\times 2\sqrt{7}$ h-BN as the top and bottom layers and 4 × 4 WS_2_ with antisite W_S_^0^ as the middle layer. The bottom h-BN layer isolates the qubit layer from the substrate, while the top h-BN layer provides protection against external environmental effects. **c** The in-gap defect levels where two electronic levels are occupied by spin-up electrons in a triplet ground state. **d** Computed density of states of the heterojunction and the projected density of states on B and N atoms indicating that the qubit can be optically initialized and readout without significant perturbation from the h-BN isolation/protection layers owing to the very large bandgap of h-BN and the type-I band alignment of h-BN and WS_2_.

Our antisite qubits, which involve TMD monolayers will be susceptible to the presence of molecules, ions, and other chemical species in the environment. In order to address this problem, we have investigated a qubit protection scheme (Fig. 4a) in which the MX_2_ monolayer is capped on both sides with a layer of hexagonal boron nitride (h-BN) as a protective cover. Based on our first-principles computations using the hybrid functional with the standard mixing parameter and the inclusion of van der Waals corrections (optPBE-vdW) for structural relaxation^48,49^, we find that the triplet ground state is preserved for the antisite qubit in the h-BN/WS_2_/h-BN heterojunction (Fig. 4b). Energy separation between the highest occupied and the lowest unoccupied defect level in the spin-up channel (related to the ZPL energy) is around 1.1 eV, which is close to that in the monolayer system. A small level splitting of 0.045 eV is observed between the two occupied defect levels, which is associated with the slight symmetry breaking induced by the neighboring h-BN layer (Fig. 4c).

The preceding observations indicate the effectiveness of adopting h-BN as protection layers to isolate the antisite qubits in monolayer TMDs from both environmental and substrate effects. As shown in Fig. 4d, the projected density of states (PDOS) on h-BN layers is located deep in the conduction and valence bands of the heterojunction due to the large bandgap of h-BN (~ 6 eV) and the type-I band alignment between h-BN and WS_2_^50^. We thus expect that the key optical transitions related to the qubit operation will not be significantly affected by the h-BN isolation/protection layers. The protected antisite qubits in 2D heterostructures thus offer a promising and robust platform for quantum information technologies.

Another key factor concerns the spin decoherence time *T*_*2*_ of a qubit. Taking MoS_2_ as an example, the previous work^24^ has shown that the decoherence of the electron spin originates mainly from the presence of ^95^Mo and ^97^Mo cation nuclear spins of 5/2, and that it can be greatly diminished by utilizing nuclear-spin-free isotope for which an exceptionally long spin decoherence time (more than 30 ms) has been predicted, owing to molybdenum’s small gyromagnetic ratio. The gyromagnetic ratio (^183^W)/(^95^Mo) is 0.64, and therefore, even longer spin decoherence time can be expected in WS_2_ and WSe_2_ based defect qubits for realizing controllable multi-qubit operations in solid-state 2D systems, decoherence effects generated by impurities and other defects notwithstanding.

## Discussion

Using a high-throughput materials discovery effort based on a defect-qubit design hypothesis involving the interplay of local symmetry of the defect and the electronic structure of the host, we identify thermodynamically stable, neutral anion-antisite defects in six monolayer 1H-MX_2_ TMD compounds as potential defect-spin qubits hosting stable triplet ground states. The optical signatures of these qubits, including the ZPLs for optical transitions, are evaluated using an in-depth analysis of the electronic configurations and the corresponding symmetry representations of the defect states in the antisites. Intersystem-crossing channels for qubit initialization and operation are identified. A scheme for isolating and protecting the antisite qubits is proposed based on a h-BN/TMD/h-BN heterojunction structure. Our study opens a new pathway for creating spin-qubits and multi-qubit platforms for quantum information technologies based on defects in 2D solid-state systems.

## Methods

### Computational details

All calculations were performed by using the Vienna Ab initio Simulation Package (VASP)^51^ based on the density functional theory (DFT)^52,53^. To calculate the spin density near the nuclei, the projector-augmented-wave method (PAW)^54,55^ and a plane-wave basis set were used. Recent advances using hybrid functionals have led to accurate descriptions of defect states by overcoming the well-known bandgap problem of the traditional DFT. Our calculations were performed using the screened hybrid-functional of Heyd-Scuseria-Ernzerhof (HSE)^56,57^ with default mixing parameter and the standard range-separation parameter (0.2 ${{{{{{\rm{\AA }}}}}}}^{-1}$) to reproduce the experimental quasiparticle gap of pristine WS_2_^39^. For defect supercell calculations, we used the $\Gamma$ point in the Brillouin zone for defect-state calculations to avoid undesirable splitting of defect states. For charged-defect formation energy calculations, a special k-point at (0.25, 0.25, 0) in the first Brillouin zone was used (Supplementary Note 3). A vacuum space of 20 Å was added along the direction perpendicular to the monolayer with a planar supercell of $5\times 5$, in order to avoid interactions between the adjacent images. Structural relaxations have been performed for all the systems investigated which were converged until the force acting on each ion was less than 0.01 eV/Å. The convergence criteria for total energies for structural relaxations and self-consistent calculations are ${10}^{-4}{{{{{\rm{eV}}}}}}$ and ${10}^{-5}{{{{{\rm{eV}}}}}}$, respectively. The constrained DFT (CDFT) methodology^58,59^ was employed for the calculation of excitation energies between the triplet states. The spin-orbit coupling is implemented as perturbation treatment in VASP^60,61^, and the out-of-plane direction is chosen as the quantization axis.

### Defect formation and transition levels

The relative stability of point defects depends on the charge states of the defects. We analyzed the stability of antisite defects in TMDs by calculating the defect formation energy (*E*_*f*_) for charge state q, which is defined as: *E*_*f*_ *(ϵ*_*F*_*) = E*_*tot*_^*q*^ *– E*_*bulk*_ + *μ*_*X*_ *– μ*_*M*_ + *q*(*ϵ*_*F*_ + *E*_*V*_) + *ΔE*, where *E*_*tot*_^*q*^ is the total energy of the charged-defect system with charge *q*, *E*_*bulk*_ is the total energy of the perfect MX_2_ system, *μ*_*M*_ is the chemical potential of the metal atom M, *μ*_*X*_ is the chemical potential of the anion atom X, *ϵ*_*F*_ is the position of the Fermi-level with respect to the valence band maximum *E*_*V*_, and *ΔE* is the charge-correction energy. Transition levels are defined as (*q*’/*q*) = (*E*_*f*_^*q’*^-*E*_*f*_^*q*^)/(*q*-*q*’), where *E*_*f*_^*q*^ is the formation energy for the state of charge *q*. We can interpret the transition levels as the Fermi level positions at which the formation energies of the defects in two distinct charge states are equal. The ionized energy of donor/acceptor is defined as the energy difference of transition level (*+*/*0*)/(0/*-*) and CBM/VBM. In a low-dimensional system, due to anisotropic screening, ionization energy (IE) diverges with respect to the vacuum, and we applied a charge-correction method^32,33^. We assume that the chemical potentials of M and X are in thermal equilibrium with MX_2_, i.e., $\mu_{{MX}_{2}}=\mu_{M}+2\mu_{X}$, where $\mu_{{MX}_{2}}$ is the energy of the perfect MX_2_ system. The accessible range of *μ*_*M*_ and *μ*_*X*_ can be further limited by the lowest energy phases of these elements depending on growth conditions. It should be noted that the transition levels do not depend on the choice of chemical potentials.

## Supplementary information

Supplementary Information

## Data Availability

The authors declare that the main data supporting the findings of this study are available within the paper and its Supplementary files. Other relevant data are available from the corresponding author upon reasonable request.
