# Supplementary material for: Antisite defect qubits in monolayer transition metal dichalcogenides
Source: Nat Commun. 2022 Jan 25;13:492. doi: 10.1038/s41467-022-28133-x (PMC8789810; doi:10.1038/s41467-022-28133-x)
Supplement: Supplementary file 1 — Supplementary Information [file 41467_2022_28133_MOESM1_ESM.pdf]

Supplementary Information

Antisite defect qubits in monolayer transition metal dichalcogenides

Jeng-Yuan Tsai[1], Jinbo Pan[1], Hsin Lin[2], Arun Bansil[3]*, Qimin Yan[1]*

[1]Department of Physics, Temple University, Philadelphia, PA 19122, USA

[2]Institute of Physics, Academia Sinica, Taipei, Taiwan

[3]Physics Department, Northeastern University, Boston, MA 02115, USA

* Correspondence and requests for materials should be addressed to A.B. (ar.bansil@northeastern.edu) and Q.Y. (qiminyan@temple.edu)

- **Supplementary Note 1, Supplementary Table 1, Supplementary Figure 1**
  Anion vacancies with 2- charge states in MX_2 (M: Mo, W; X: S, Se, Te)

- **Supplementary Note 2, Supplementary Table 2**
  Pristine and defective 1H-TMDs

- **Supplementary Note 3, Supplementary Figure 2**
  Transition levels and ionization energies of anion antisites in 1H-TMDs MX_2 (M: Mo, W; X: S, Se, Te)

- **Supplementary Note 4, Supplementary Figure 3**
  Antisite defect qubit in WSe_2

- **Supplementary Note 5, Supplementary Figure 4, Supplementary Tables 3-5**
  Zero-phonon line and zero-field splitting for M_X^0 (M: Mo, W; X: S, Se)

- **Supplementary Note 6, Supplementary Figure 5**
  Antisite qubit W_S^0 with local-symmetry C_h

- **Supplementary Note 7, Supplementary Figure 6**
  Qubit operation of W_S^0 antisite with local-symmetry C_h

- **Supplementary Note 8**
  Symmetry-allowed intersystem crossings in W_S^0

- **Supplementary Note 9, Supplementary Table 6**
  Formation energies for M_X^0, X_X^0, V_X^0, and V_M^0 (M: W; X: S, Se)

- **Supplementary Note 10, Supplementary Figure 7**
  Correlation between the displacement in the *z*-direction and the defect-level pattern

- **Supplementary Note 11, Supplementary Figure 8**
  Correlation between the equilibrium *z*-positions of the antisites and anion species

- **Supplementary Note 12, Supplementary Figure 9**
  Defect levels of M_X^0 (M: Mo, W; X: S, Se, Te) with spin-orbit coupling.

- **Supplementary Note 13, Supplementary Tables 7-9**
  Calculated ZPLs with SOC for different occupation configurations in CDFT.

Supplementary Note 1: Anion vacancies with 2- charge states in MX_2 (M: Mo, W; X: S, Se, Te).

We performed a set of calculations for anion vacancies in TMD monolayers in 2- charge state with an even number of electrons. Energy differences between the triplet and singlet states, listed in Supplementary Table 1, indicate that these negatively charged vacancy defects host triplet ground states. Despite the favorable energetics, the defect levels for all these negatively charged vacancies are fully occupied in the spin-up channel, so that the only plausible transitions lie within the spin-down channel (Supplementary Figure 1). Unfortunately, all the unoccupied defect levels in the spin-down channel lie at fairly high energies due to the exchange interactions and locate in the conduction bands. This lack of unoccupied in-gap defect levels makes the necessary qubit excitation process impossible. We thus rule out the negatively charged anion vacancy as a plausible qubit candidate.

Defect	$\Delta E_{triplet-singlet}$ (eV)
V_S^{2-} in MoS_2	-0.179
V_{Se}^{2-} in $MoSe_2$	-0.193
V_{Te}^{2-} in $MoTe_2$	-0.146
V_S^{2-} in WS_2	-0.130
V_{Se}^{2-} in WSe_2	-0.140
V_{Te}^{2-} in WTe_2	-0.196

Supplementary Table 1: Energy differences between the triplet and singlet states of anion vacancies with 2- charge states. The negative energy differences between the triplet and singlet states of anion vacancies V_X^{2-} in MX_2 (M: Mo, W; X: S, Se, Te) indicate that these negatively charged vacancies host stable triplet ground states.

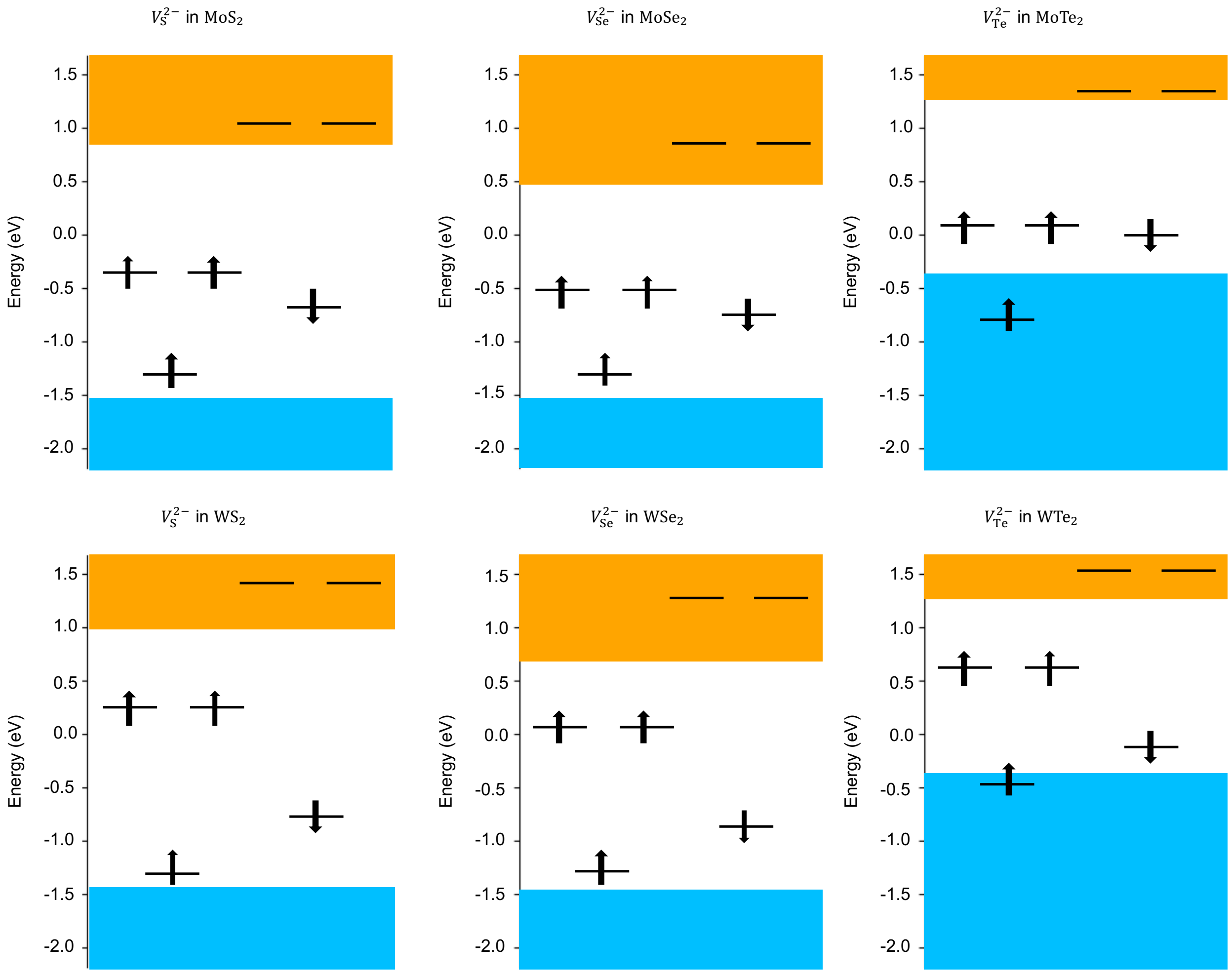

Supplementary Figure 1: Schematic defect-level diagrams of anion vacancies with 2- charge states. The defect levels are comprised by fully occupied a_1 level and half-occupied e levels. The unoccupied e levels in spin-down channel lie in the conduction bands, which makes defect-level-based optical transitions in the spin-down channel impossible.

Supplementary Note 2: Pristine and defective 1H-TMDs.

The calculated geometric and electronic structure related data for pristine and defective 1H-TMDs (with *C_{3v}* local symmetry) are presented in Supplementary Table 2.

Pristine TMD	WS_2	WSe_2	WTe_2	MoS_2	$MoSe_2$	$MoTe_2$
a (Å)	3.147	3.275	3.505	3.147	3.278	3.505
d_{M-X} (Å)	2.391	2.513	2.701	2.385	2.511	2.700
d_{X-X}(Å)	3.107	3.311	3.578	3.090	3.300	3.573
Antisite M_X^0	W_S^0	W_{Se}^0	W_{Te}^0	Mo_S^0	Mo_{Se}^0	Mo_{Te}^0
d_{A-M}(Å)	2.633	2.615	2.578	2.669	2.655	2.579
$d_{A-X_{below}}$(Å)	3.516	3.596	2.916	3.549	3.632	3.065
d-orbital order	$d_{xy}, d_{x^2-y^2}$, d_{z^2}	$d_{xy}, d_{x^2-y^2}$, d_{z^2}	$d_{z^2}, (d_{xy}, d_{xz})$ $(d_{yz}, d_{x^2-y^2})$	$d_{xy}, d_{x^2-y^2}$, d_{z^2}	$d_{xy}, d_{x^2-y^2}$, d_{z^2}	$d_{z^2}, (d_{xy}, d_{xz})$ $(d_{yz}, d_{x^2-y^2})$
$\Delta E_{triplet-singlet}$ (eV)	-0.273	-0.295	-0.301	-0.349	-0.343	-0.264

Supplementary Table 2: Geometric parameters for pristine MX_2 and neutral anion antisites M_X^0 (M: Mo, W; X: S, Se, Te). d_{M-X} denotes the distance between a cation and its adjacent anions. d_{X-X} is the distance between anions in the upper and lower anion layers. In the defective systems, the distances between the antisite (labeled A) and the adjacent cations M, are labeled by d_{A-M}. The distances between the antisite cation A and the anion in the bottom layer are labeled by $d_{A-X_{below}}$(Å). The *d*-orbital order refers to the increased ordering of in-gap defect levels in energy. Defect levels are mainly contributed by *d*-orbitals of the antisite. M_{Te}^0 in $MoTe_2$ and WTe_2 hosts a set of levels labeled as d_{z^2}, (d_{xy}, d_{xz}), and $(d_{yz}, d_{x^2-y^2})$, where brackets represent the hybridization of *d*-orbitals.

Supplementary Note 3: Transition levels and ionization energies of anion antisites in 1H-TMDs MX_2 (M: Mo, W; X: S, Se, Te).

The anisotropic-correction method for charged defect systems is based on the extrapolation of an asymptotic expression of ionization energy (IE):[1,2] $\mathrm{IE}(S, Lz) = IE_0 + \frac{\alpha}{\sqrt{S}} + \frac{\beta}{S} L_z$, where IE_0 is size-independent ionization energy, S is the area of the 2D system, and L_z is the vacuum thickness. Here, α is the Madelung constant for a point charge and mainly depends on geometry instead of the defect type.[1,2] Supercells were built with sizes of 5×5 and 6×6 and vacuum thicknesses of 20 and 25 Å. To test the convergence of extrapolation, we performed an additional set of calculations for the antisite defect W_S in WS_2 with a supercell size of 4x4. Differences in the ionization energies between what is fitted by 3-size calculations (4x4, 5x5, and 6x6) and 2-size calculations (5x5 and 6x6) are 0.14 eV and 0.05 eV for the donor and acceptor states, respectively. This level of differences does not affect any of the main conclusions of this study. The charge states q = -1, 0, and 1 were considered for all anion antisites. The anisotropic correction was carried out in two steps: (1) We extrapolated $\mathrm{IE}(S, L_z)$ with respect to L_z. The y-intercept of the linear line corresponds to $IE_0 + \frac{\alpha}{\sqrt{S}}$. Different lines are the extrapolations based on different sizes of supercells. This was followed by step (2) where we extrapolated $IE_0 + \frac{\alpha}{\sqrt{S}}$ with respect to $\frac{1}{\sqrt{S}}$. Here the y-intercept is the size-independent ionization energy IE_0. The ionization energy of the donor-state $\epsilon(+/0)$ /acceptor-state $\epsilon(0/-)$ is defined as the energy difference between the donor state/acceptor state and CBM/VBM.

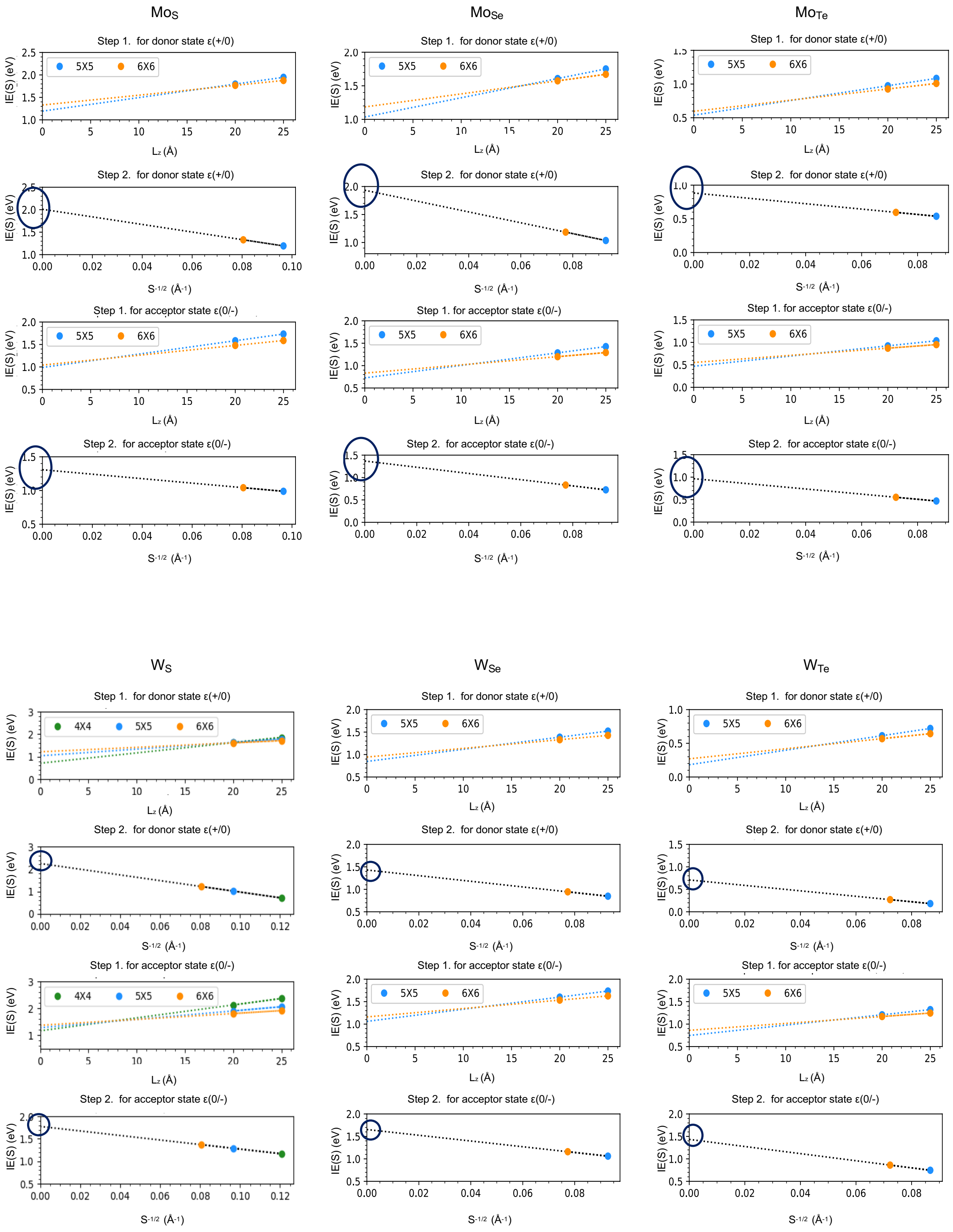

Supplementary Figure 2: Implementation of extrapolation method for anisotropic corrections of low-dimensional charged defects. Black circles highlight the size-independent

ionization energies (IE_0) of the donor $\epsilon(+/0)$ and acceptor states $\epsilon(0/-)$ for various anion antisites. W_S: (2.24 eV, 1.77 eV), W_{Se}: (1.43 eV, 1.65 eV), W_{Te}: (0.71 eV, 1.43 eV), Mo_S: (2.01 eV, 1.31 eV), Mo_{Se}: (1.93 eV, 1.37 eV), and Mo_{Te}: (0.88 eV, 0.97 eV). To test the convergence of extrapolation, extra data points obtained from 4x4 supercell calculations are included in the case of W_S^0.

Supplementary Note 4: Antisite defect qubit in WSe_2.

Antisite qubit W_{Se}^0 in WSe_2 resembles W_S^0 in WS_2 in terms of defect geometry and electronic structure. Optimized pristine structure is characterized by the W-Se and Se-Se distances of 2.513 Å and 3.311 Å, respectively. Hybrid functional calculations predict a band gap of 2.13 eV, which is comparable with experimental result.[3] The calculated in-gap defect levels, labeled by C_{3v} point group, are shown in Supplementary Figure 3b. Optical transitions were calculated with consideration of SOC, and the ZPL is 0.585 eV. Position of the singlet ${}^1A_1$ is 0.382 eV above ${}^3A_2$, which is estimated by considering the Coulombic interaction. Note that the energy difference between ${}^3A_2$ and ${}^1E$ was calculated with consideration of SOC. The zero-field splitting (ZFS) between the sublevels E and A_1 in the triplet ground state is found to be 6.81 GHz (Supplementary Note 5). Nonradiative decay paths are identified based on group theory and are identical to the case of W_S^0 in WS_2.

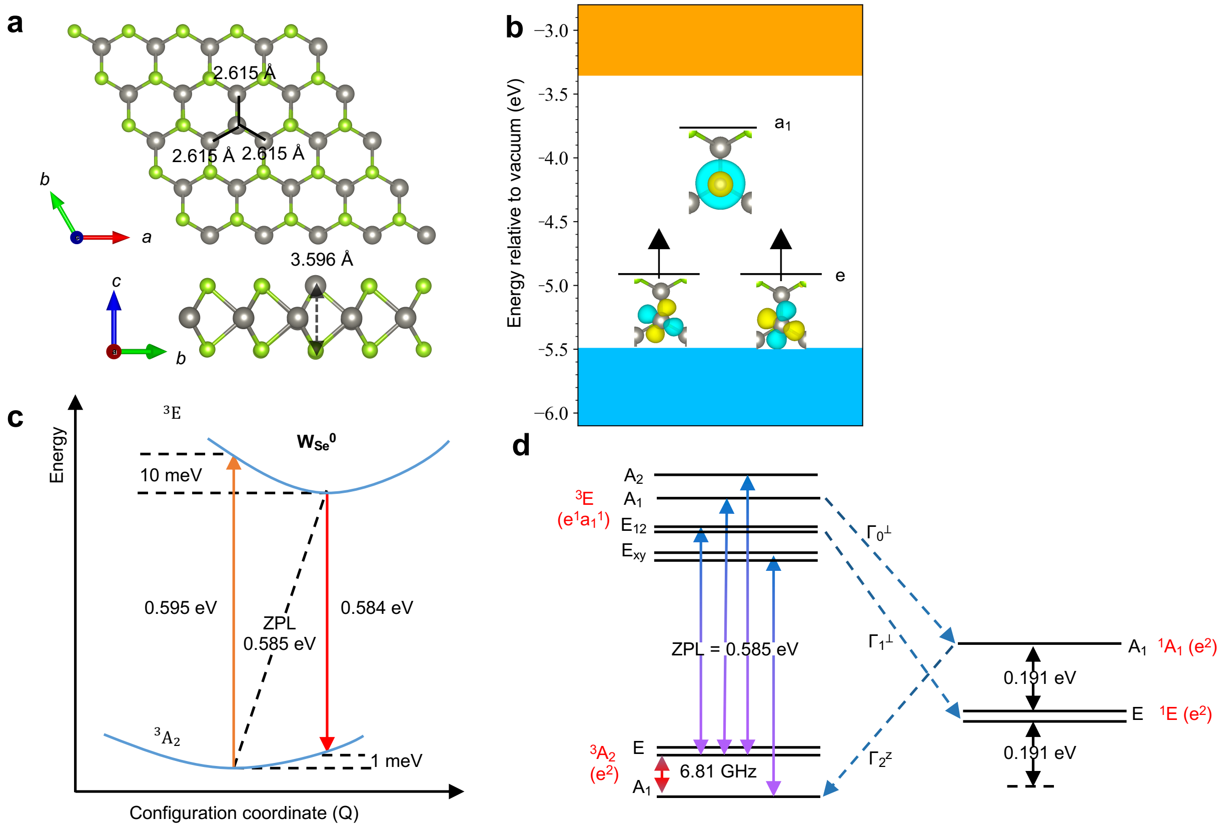

Supplementary Figure 3: Geometry and electronic structures of neutral antisite qubit W_{Se}^0 in WSe_2. **a** Optimized structure of W_{Se}^0 antisite in WSe_2. **b** Energy diagram of the defect levels in

the triplet ground state ${}^3A_2$. The defect levels e and a_1 mainly derived from $\{d_{x^2-y^2}, d_{xy}\}$ and d_{z^2} orbitals of the antisite. Wavefunctions of these three levels are shown schematically. **c** Configuration coordinate diagram of W_{Se}^0 in WSe_2 for the triplet ground state ${}^3A_2$ and the triplet excited state ${}^3E$. **d** Sublevels for the triplet ground state ${}^3A_2$, the triplet excited state ${}^3E$, and the singlet states ${}^1E$ and ${}^1A_1$ (labeled by the IRs of C_{3v}). Spin-conserving optical transitions are shown by colored solid arrows. Symmetry-allowed intersystem-crossing paths are denoted by dashed arrows. The labels $\{\Gamma_0^{\perp}, \Gamma_1^{\perp}\}$ and Γ_2^z indicate the allowed intersystem-crossing paths via nonaxial spin-orbit coupling and axial spin-orbit coupling, respectively.

Supplementary Note 5: Zero-phonon line and zero-field splitting for M_X^0 (M = Mo, W; X = S, Se).

Optical transitions and the ZFS of the sublevels play a key role in the operation and robustness of a defect qubit. We have computed these transitions for our four qubit candidates M_X^0 (M = Mo, W; X = S, Se), including the zero-phonon lines (ZPLs) and relaxation energies, both with (Supplementary Table 3) and without spin-orbit coupling (Supplementary Table 4) using the hybrid functional HSE06 and constrained DFT. Notations in Supplementary Tables 3-5 are defined in Supplementary Figure 4. ZFSs of the sublevels in the triplet ground states for M_X^0 (M = Mo, W; X=S, Se) due to the spin-spin dipolar coupling were evaluated by spin-spin dipolar Hamiltonian, $\hat{H}_{SS} = \hat{\mathbf{S}}\mathbf{D}\hat{\mathbf{S}}$, where $\hat{\mathbf{S}}$ is the total-spin operator and $\mathbf{D}$ is a 3 × 3 ZFS tensor[4] (Supplementary Table 5). In systems with axial symmetry such as C_{3v}, $\hat{H}_{SS}$ can be expressed as $\hat{H}_{SS} = D(S_z^2 - \frac{S(S+1)}{3})$, where $D = \frac{3}{2}D_{zz}$ is a measurable quantity, S_z is the spin projection along the z-direction, and S is the total spin. The modified Python package Pyzfs[5] was used to calculate the scalar parameter D, which involves the use of pseudo-wavefunctions obtained from the VASP code. Previous calculations for NV^- center in diamond based on this method give a ZFS of 2.90 GHz[6] and 2.854 GHz[7], which are close to the experimental value of 2.88 GHz.[8]

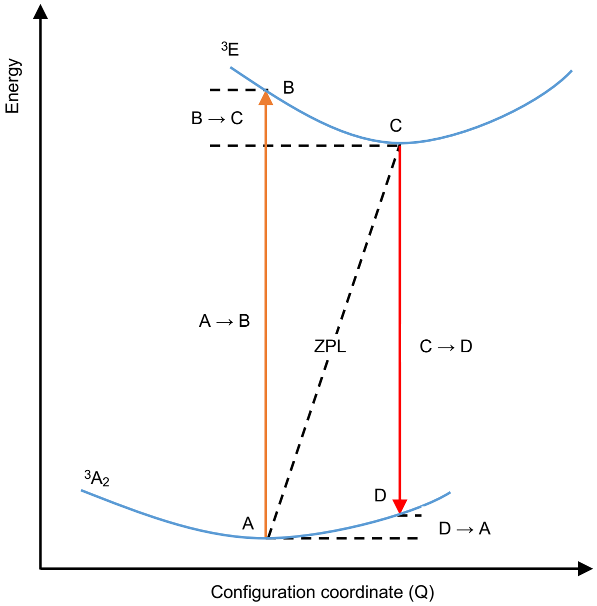

Supplementary Figure 4: Notations for optical transitions and relaxation energies. A → B and C → D represent vertical transitions between the ground and excited states. B → C and D → A represent the Franck-Condon relaxation in the ground and excited state, respectively.

Defect	$A \rightarrow B$ (eV)	$B \rightarrow C$ (meV)	$C \rightarrow D$ (eV)	$D \rightarrow A$ (meV)	ZPL (eV)
W_S^0	0.732	5	0.726	1	0.727
W_{Se}^0	0.595	10	0.584	1	0.585
Mo_S^0	0.932	1	0.930	1	0.931
Mo_{Se}^0	0.817	3	0.813	1	0.814

Supplementary Table 3: Optical transition energies and relaxation energies calculated using the hybrid functional HSE06 with spin-orbit coupling.

Defect	$A \rightarrow B$ (eV)	$B \rightarrow C$ (meV)	$C \rightarrow D$ (eV)	$D \rightarrow A$ (meV)	ZPL (eV)
W_S^0	0.738	4	0.733	1	0.734
W_{Se}^0	0.648	11	0.635	2	0.637
Mo_S^0	0.890	3	0.886	1	0.887
Mo_{Se}^0	0.804	4	0.800	1	0.801

Supplementary Table 4: Optical transition energies and relaxation energies calculated using the hybrid functional HSE06 without spin-orbit coupling.

Defect	Zero-field splitting D (GHz)
Mo_S^0	8.41
Mo_{Se}^0	7.22
W_S^0	7.89
W_{Se}^0	6.81

Supplementary Table 5: Zero-field splitting parameters for the triplet ground states $^3A_2$ in M_X^0 (M = Mo, W; X = S, Se).

Supplementary Note 6: Antisite qubit W_S^0 with local-symmetry C_h.

If a random symmetry-breaking structural perturbation is applied, W_S^0 antisite may experience an in-plane displacement from the center, which would lower the symmetry from C_{3v} to C_h. Note that lowering C_{3v} to C_h reduces the total energy by only about 25 meV per unit cell. The results of W_S^0 with local symmetry C_h are presented in this section. Symmetry-lowering causes the splitting of the degenerate sublevels S_x ($m_s = 1$) and S_y ($m_s = -1$). We evaluated this splitting by calculating the **D** tensor due to spin-spin dipolar interaction. The scalar parameters D and E defined as $\frac{3}{2}D_{zz}$ and $\frac{D_{xx}-D_{yy}}{2}$ describe the splitting between $m_s = \pm 1$ and $m_s = 0$ and the splitting between $m_s = 1$ and $m_s = -1$, respectively. The calculated $|D|$ and $|E|$ values are 6.60 GHz and 1.25 GHz, respectively. The ratio of $|D|$ and $|E|$ is about 5:1, implying that the extent of deviation from C_{3v} to C_h is relatively small.

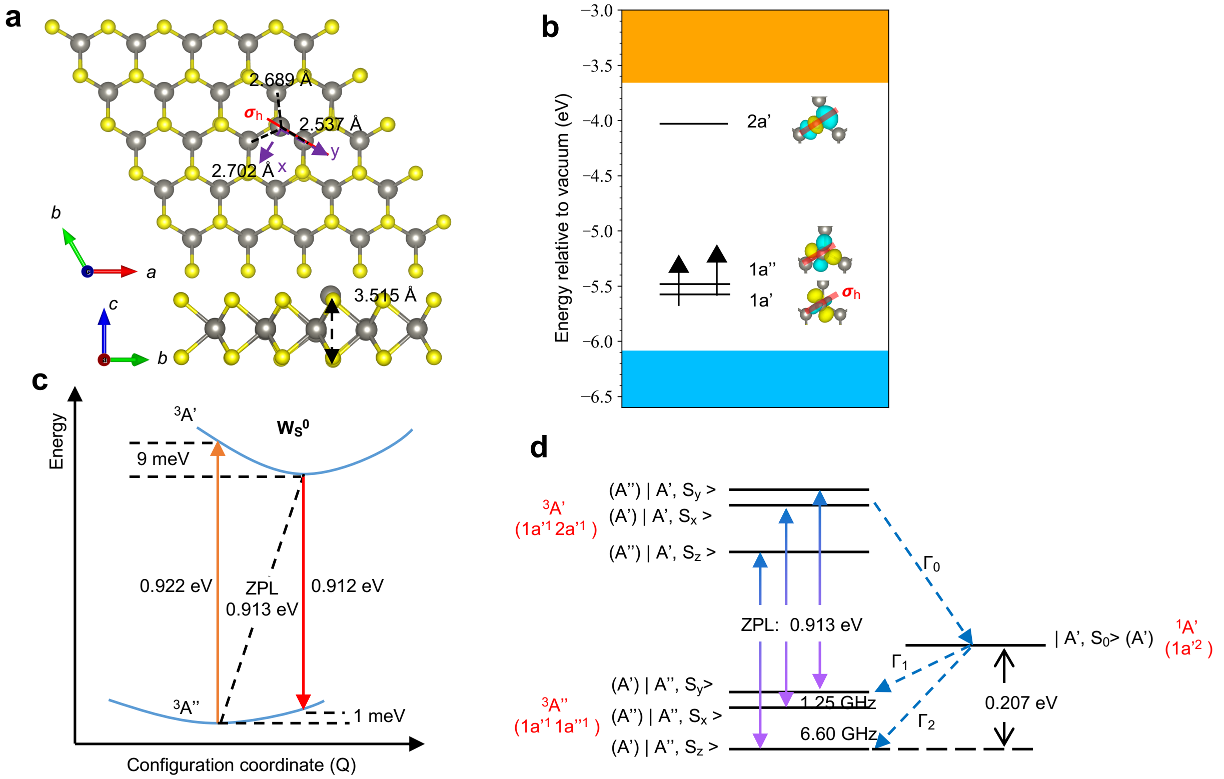

Supplementary Figure 5: Geometric and electronic structure of neutral antisite defect W_S^0 in WS_2 with local-symmetry C_h. **a** The relaxed structure of W_S^0 with local-symmetry C_h. The y-direction is defined to lie along the mirror plane σ_h (red line) and the x-direction is perpendicular to σ_h . **b** A schematic energy diagram of the in-gap defect levels and the depictions of

wavefunctions. Defect levels are labeled by $1a'$, $1a''$, and $2a'$ from low to high energy. Despite hybridization with the neighboring d-orbitals, the lowest unoccupied level is mainly contributed by d_{z^2} orbital of the antisite. **c** Calculated configuration coordinate diagram with the zero-phonon-line (ZPL) of 0.913 eV and Frank-Condon relaxation energies of 9 meV and 1 meV for the excited and ground state, respectively. The optical transitions were calculated with consideration of SOC. **d** Sublevels and the corresponding IRs for the triplet ground state $^3A''$, triplet excited state $^3A'$, and the singlet state $^1A'$. The singlet state $^1A'$ is 0.207 eV above $^3A''$ with consideration of SOC. Due to symmetry-lowering, the sublevels $m_s = \pm1$ have a relatively small splitting of 1.25 GHz, compared to the splitting of 6.60 GHz between $m_s = 1$ and $m_s = 0$. The allowed intersystem-crossing paths are denoted by Γ_0, Γ_1, and Γ_2, where Γ_1 is a weak transition due to the slightly lowered symmetry from C_{3v} to C_h.

Supplementary Note 7: Qubit operation of W_S^0 antisite with local-symmetry C_h.

According to the discussion in Supplementary Note 6, an approximate two-level qubit system can be formed by the sublevels $m_s = \pm 1$ and $m_s = 0$. A key difference between the defect qubit in C_{3v} and C_h symmetries is reflected in the relative strengths of the intersystem-crossing paths Γ_1 and Γ_2. For large $|E|$ (see Supplementary Note 6 for the definition of E), the system is in C_h symmetry and it deviates dramatically from C_{3v}, resulting in a strong Γ_1 and a weak Γ_2. Although Γ_1 allowed in our case, its strength is thus quite small. As a result, during initialization and readout, only a small fraction of the population will be polarized into the sublevel S_y ($m_s = -1$), and a majority of the population will occupy the sublevel S_z ($m_s = 0$) via the strong intersystem-crossing path Γ_2.

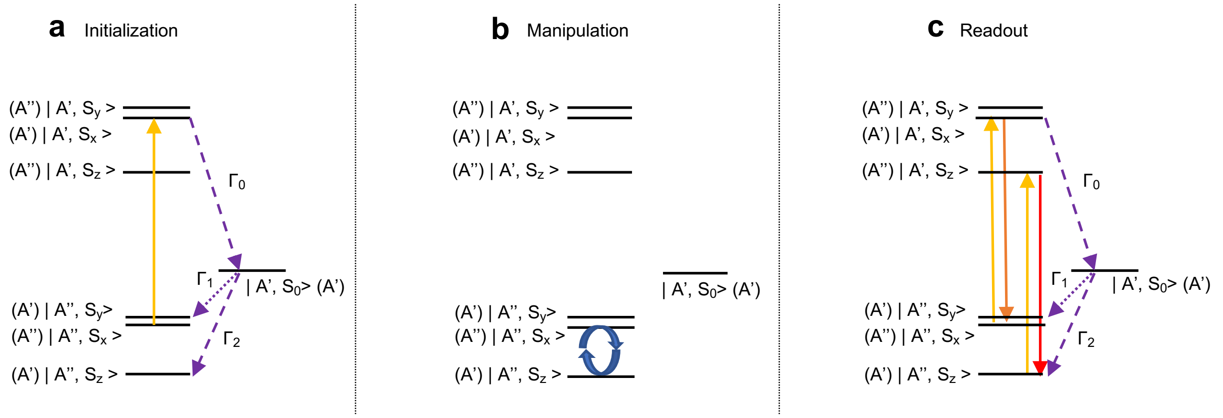

Supplementary Figure 6: Operational loop for W_S^0 antisite qubit with local-symmetry C_h. a Initialization of the qubit by pumping the system optically. Due to the existence of the intersystem paths Γ_0 and Γ_2, most of the population is polarized into the sublevel S_z. Note that Γ_1 is a weak transition because the symmetry is lowered only slightly from C_{3v} to C_h. **b** Manipulation of the qubit can be implemented via techniques similar to those used in NV^- center such as electron paramagnetic resonance (EPR). **c** Readout process can also be implemented in analogy with NV^- by pumping the antisite first and then detecting intensity differences in luminescence. Note that the luminescence involving $m_s = \pm 1$ is weaker than that involving $m_s = 0$ due to the existence of the intersystem crossing path Γ_0.

Supplementary Note 8: Symmetry-allowed intersystem crossings in $\mathbf{W_S^0}$.

The single-particle spin-orbit coupling operator $\hat{H}_{SO}$ with C_{3v} symmetry can be defined as $\hat{H}_{SO} = \sum_k \lambda_{xy}\left(l_k^x s_k^x + l_k^y s_k^y\right) + \lambda_z l_k^z s_k^z$, where l_k^i and s_k^i denote the angular momentum and spin operator projected onto the $\mathrm{i^{th}}$ component for the $\mathrm{k^{th}}$ electron, respectively.[9] The nonaxial and axial strengths of spin-orbit coupling are denoted by λ_{xy} and λ_z, respectively. If the system is imposed by axial symmetry, one can rewrite $\hat{H}_{SO}$ in terms of the raising and lowering operators as: $\hat{H}_{SO} = \sum_k \lambda_{xy}(l_k^+ s_k^- + l_k^- s_k^+) + \lambda_z l_k^z s_k^z$, where $l_k^\pm$ and $S_k^\pm$ are the raising and lowering operators for the angular momentum and spin operator, respectively. Nonaxial components include $l^\pm$ and $s^\pm$ that mix different Slater-determinants (i.e. mix e^2 and $a_1^1 e^1$) and different spin projections. On the other hand, the axial component contains l_z and s_z that is only able to mix identical Slater-determinants and spin projections. We emphasize that $\mathrm{NV^-}$ center hosts identical electron/hole configurations (e: $a_1^2 e^2$, h: e^2) and sublevel symmetries. Note that nonaxial spin-orbit interaction is much weaker than the axial one in the $\mathrm{NV^-}$ center.[10]

Intersystem crossing is allowed only if $< \Psi_i|\hat{H}_{SO}|\Psi_f >$ is nonzero. Since $\hat{H}_{SO}$ is a scalar operator, which belongs to IR A_1, the matrix element would be non-zero only if the involved IRs satisfy the condition: $\mathrm{rep}(\Psi_i)\otimes\mathrm{rep}(\hat{H}_{SO})\otimes\mathrm{rep}(\Psi_f) \supset A_1$. For the triplet ground state, the spatial wavefunction $\psi_{spatial}$ belongs to IR A_2 and the three spin projectors $\{S_x, S_y\}$ and S_z belong to the 2D IR E and 1D IR A_2, respectively. By decomposing the reducible representation $(E \oplus A_2)\otimes A_2$ into the sum of IRs in C_{3V}, we obtain the 2D degenerate sublevels E and the 1D sublevel A_1. The sublevel space forms a 3D state space that is given by the product of the dimensions of $\psi_{spatial}$ (1D) and the spin projectors (3D). Accordingly, for the triplet excited state ${}^3E$, we have a six-dimensional sublevel space consisting of IRs $\{E_{12}, E_{xy}, A_1, A_2\}$. Here, the IRs $\{E_{12}, A_1, A_2\}$ involves spin components $\{S_x, S_y\}$ while the IR E_{xy} only involves S_z. The singlet states ${}^1E$ and ${}^1A_1$ form sublevels labeled by E and A_1, which share a common spin-zero component (S_0). A nonzero matrix element also depends on the condition that the axial (nonaxial) spin-orbit operators mix sublevels with identical (distinct) Slater-determinants and spin projectors. As a result, we identified three symmetry-allowed intersystem crossings as noted in the main text.

Supplementary Note 9: Formation energies for $\mathbf{M_x^0}, \mathbf{X_x^0}, \boldsymbol{V_x^0}$, and $\boldsymbol{V_M^0}$ (M: W; X: S, Se).

The formation energy of a neutral defect is defined as: $E_f = E_{tot}(\text{defect}) - E_{tot}(\text{MX}_2) - \sum_\text{i} n_i\mu_i$, where n_i is a positive/negative integer when adding/removing an atom. For tungsten, the adopted reference phase is the bulk metallic body-centered (bcc) structure. For sulfur and selenium, S_8 and Se_8 allotropes were used. Note that the formation energies of defects depend on experimental growth conditions. For this reason, we consider both W-rich and X-rich conditions and find that the anion antisites have the second-lowest energies under W-rich conditions. The relatively high formation energies indicate that the intrinsic concentration of antisite defects in the as-grown TMDs under equilibrium conditions would be low. Anion antisites could potentially also be created under nonequilibrium conditions involving irradiation or ion implantation. Note that anion antisite defects in both WS_2 and WSe_2 have been observed experimentally.[11,12]

Defect in WS_2	W-rich (eV)	S-rich (eV)	Defect in WSe_2	W-rich (eV)	Se-rich (eV)
W_S^0	5.45	9.44	W_{Se}^0	5.60	8.47
S_W^0	9.75	5.75	Se_W^0	7.59	4.72
V_W^0	7.73	5.07	V_W^0	6.42	4.50
V_S^0	1.63	2.96	V_{Se}^0	1.94	2.90

Supplementary Table 6: Formation energies for neutral anion antisites ($\mathbf{M_X^0}$), neutral cation antisites ($\mathbf{X_M^0}$), neutral anion vacancies ($\boldsymbol{V_X^0}$), and neutral cation vacancies ($\boldsymbol{V_M^0}$) (M: W; X: S, Se) under W-rich and X-rich conditions.

Supplementary Note 10: Correlation between the displacement in the z-direction and the defect-level pattern.

In order to gain insight into the differences in level-splitting patterns in various 1H-TMDs, we performed an analysis of their bonding using the crystal orbital Hamilton population (COHP) approach[13]. In this way, we identified a key difference in the orbital interactions that is driven by the z-position of the antisite. Results for anion antisites in WS_2 for different z-positions are shown in Supplementary Figure 7. As the antisite moves toward the cation plane, a_1 and e levels form two overlap patterns involving the wavefunctions of the antisite and the adjacent cations. In regime 1, the a_1 (mainly d_{z^2}) as well as the e level (mainly $(d_{xy}, d_{x^2-y^2})$) displays an enhanced overlap, and both the in-gap a_1 and e levels move up in energy due to their antibonding nature identified via the COHP analysis. In regime 2, the wavefunction overlap associated with the e level continues to increase as the antisite moves toward the cation plane, but the overlap of the a_1 level becomes weaker. As the two sets of defect levels get closer in energy with the antisite moving toward the cation plane, at z = -0.3 Å, a defect-level switching takes place. Note that the three levels a_1, e_1, and e_2 are occupied by two electrons, resulting in the e levels splitting into e_1 and e_2 due to the Jahn-Teller effect. It is thus the difference in wavefunction overlaps and orbital interactions which is responsible for the two level-splitting patterns discussed in the main text.

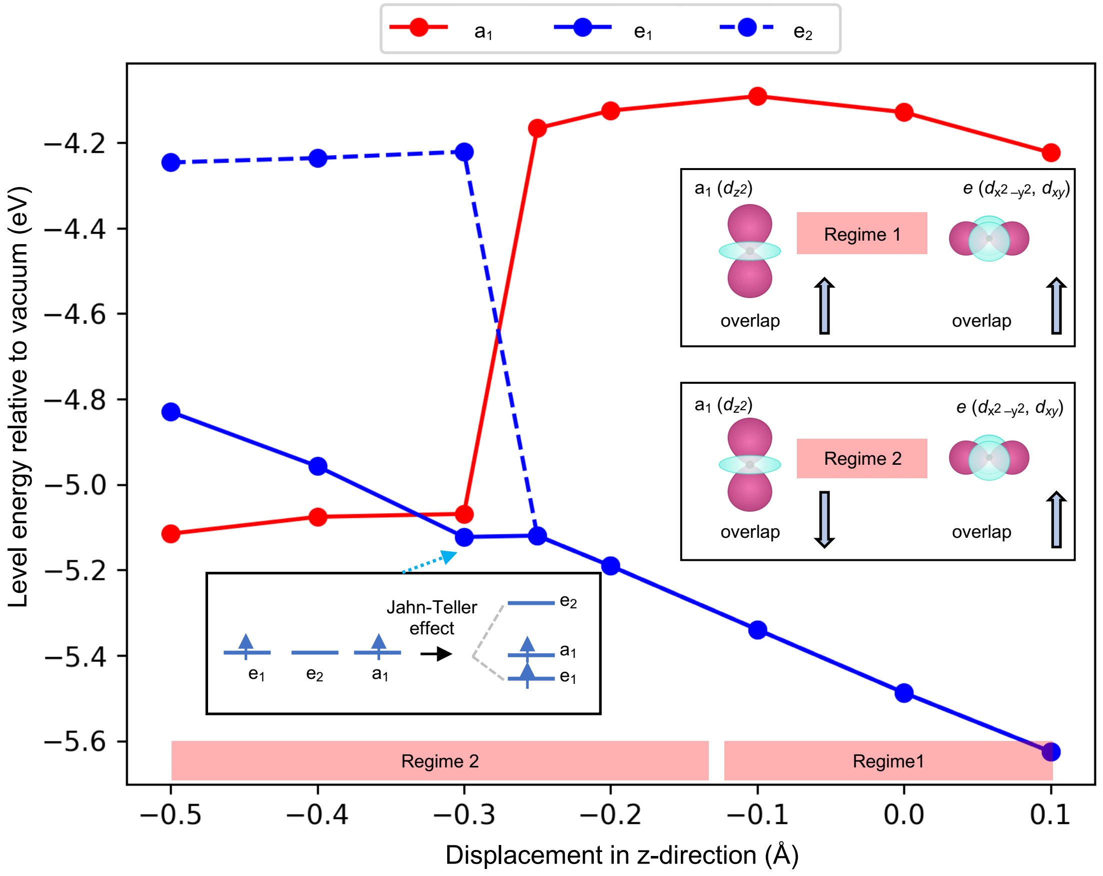

Supplementary Figure 7: Correlation between the displacement of the antisite in the *z*-direction and the defect-level energies. As the antisite moves toward the cation plane, the wavefunction overlap for the a_1 level increases in regime 1, while it decreases in regime 2. On the other hand, the wavefunction overlap of the *e* levels increases in both regimes. At z = -0.3, the e levels split into e_1 and e_2 due to magnetic Jahn-Teller effect. Blue lines: *e* levels, red lines: a_1 level. For the *x*-axis, zero is the equilibrium *z*-position, positive values denote moving upward relative to the equilibrium *z*-position, and negative values denote moving downward relative to equilibrium *z*-position.

Supplementary Note 11: Correlation between the equilibrium *z*-positions of the antisites and anion species.

In order to analyze the effects of different chemical environments of the antisites in S-, Se-, and Te-based TMDs, we performed a Bader charge analysis[14] and found the associated Bader volumes of the antisites to be larger than those of the chalcogen anions (S and Se), resulting in the antisites as replacements of the anions to shift up along the *z*-direction. In contrast, the Bader volume of the antisite is smaller than that of the Te anion in Te-based TMD, and as a result the antisite shifts down along the *z*-direction. The equilibrium *z*-positions of the antisites thus depend on the anion species (Supplementary Figure 8).

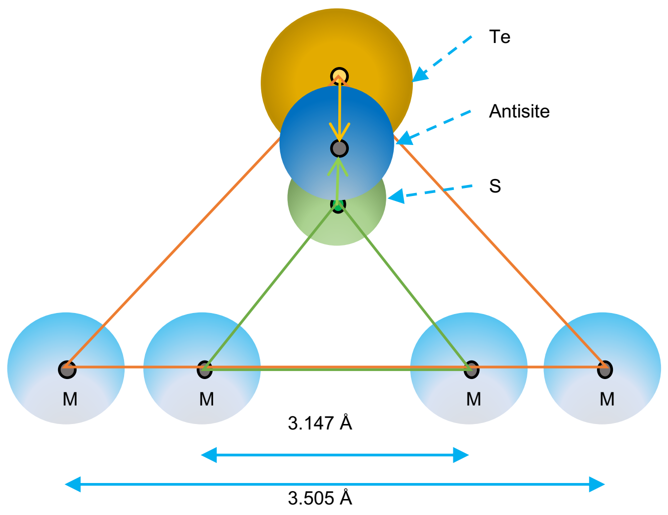

Supplementary Figure 8: A schematic illustrating the correlation between the location of the antisites and anion species S and Te. Depending on the anion (S, Te) involved, the equilibrium *z*-position of the antisite exhibits two patterns. For S, the antisite moves up to a higher equilibrium *z*-position. For Te, the antisite moves down to a lower equilibrium *z*-position. The vertical yellow arrow shows the displacement between the atomic center of antisite and Te along the *z*-direction, and the vertical green arrows shows the displacement between the atomic center of antisite and S along the *z*-direction. Note that 3.147 Å and 3.505 Å are lattice constants of WS_2 and WTe_2. M denotes the adjacent cations (Mo/W) around the antisite.

Supplementary Note 12: Defect levels of M_X^0 (M: Mo, W; X=S, Se, Te) with spin-orbit coupling.

As the nature of anion antisite defects is largely determined by the relatively massive cations in TMDs, the defect levels are affected by the spin-orbit coupling (SOC). We performed calculations in which spin-orbit interaction was treated perturbatively. For both the W- and Mo-based antisites, with the SOC, the occupied defect level *e* splits into two levels. The defect level a_1 is barely affected due to its d_{z^2} nature with zero projected angular quantum number m_l. For W_S^0 and W_{Se}^0, the energy splittings between the highest two occupied levels are estimated to be 0.374 eV and 0.343 eV, respectively. For Mo_S^0 and Mo_{Se}^0, the splittings are 0.114 eV and 0.118 eV, respectively. For the Te-based antisites, the level splittings between the lowest unoccupied and the highest occupied defect levels are 0.546 eV for W_{Te}^0 and 0.676 eV for Mo_{Te}^0.

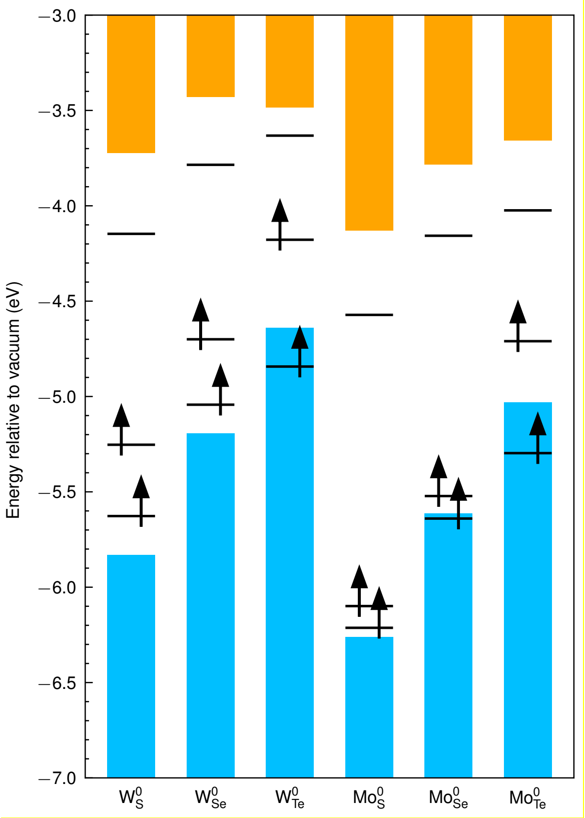

Supplementary Figure 9: Defect levels of M_X^0 (M: Mo, W; X=S, Se, Te) with the spin-orbit coupling. The defect levels are denoted by black lines and the arrows represent electrons. The numbers in parenthesis are energies relative to the vacuum. The blue and orange bars denote valence and conduction bands, respectively.

Supplementary Note 13: Calculated ZPLs with SOC for different occupation configurations in CDFT.

The ZPLs in Figure 2 (main text), Supplementary Figigures 3 and 5, and Supplementary Tables 3 and 4 are based on the excitations obtained by setting the two highest occupied levels in the ground state as half occupation and the lowest unoccupied level in the ground state as full occupation in CDFT, which is an occupation configuration that is consistent with what is used for the NV^- center in diamond. Since the highest two occupied levels of the antisite naturally split due to SOC effects, it is necessary to consider other potential excited-state occupation configurations, including the one-electron excitation from the highest occupied level of the ground state (denoted as 1-0-1) and the one-electron excitation from the second-highest occupied level of the ground state (denoted as 0-1-1) within the CDFT framework. The calculated ZPLs, vertical transitions, and Franck-Condon relaxations for these two configurations are shown in Supplementary Tables 7 and 8. For W_S^0 with local symmetry C_h, the calculated optical transition energies and relaxation energies with the 0-1-1 occupation configuration are shown in Supplementary Table 9. Notation for the optical transitions and relaxation energies are the same as in Supplementary Figure 4. The results clearly show that the ZPLs are dependent on the excited-state occupation configurations. More rigorous treatment of the excited states, such as many-body perturbation theory, is called for to provide a more accurate evaluation of the optical transitions associated with these antisite defects.

Defect	A → B (eV)	B → C (meV)	C → D (eV)	D → A (meV)	ZPL (eV)
W_S^0	0.269	4	0.264	1	0.265
W_{Se}^0	0.210	8	0.201	1	0.202
Mo_S^0	0.587	2	0.584	1	0.585
Mo_{Se}^0	0.537	4	0.532	1	0.533

Supplementary Table 7: Optical transition energies and relaxation energies calculated using the hybrid functional HSE06 with spin-orbit coupling. The excitation from the highest occupied level to the lowest unoccupied level of the ground state (denoted as 1-0-1) is considered.

Defect	$A \rightarrow B$ (eV)	$B \rightarrow C$ (meV)	$C \rightarrow D$ (eV)	$D \rightarrow A$ (meV)	ZPL (eV)
W_S^0	0.677	6	0.670	1	0.671
W_{Se}^0	0.550	11	0.538	1	0.539
Mo_S^0	0.787	7	0.779	1	0.780
Mo_{Se}^0	0.689	6	0.682	1	0.683

Supplementary Table 8: Optical transition energies and relaxation energies calculated using the hybrid functional HSE06 with spin-orbit coupling. The excitation from the second-highest occupied level to the lowest unoccupied level of the ground state (denoted as 0-1-1) is considered.

Occupation configuration	$A \rightarrow B$ (eV)	$B \rightarrow C$ (meV)	$C \rightarrow D$ (eV)	$D \rightarrow A$ (meV)	ZPL (eV)
0.5-0.5-1	0.922	9	0.912	1	0.913
1-0-1	0.496	10	0.485	1	0.486
0-1-1	0.871	10	0.860	1	0.861

Supplementary Table 9: Optical transition energies and relaxation energies for W_S^0 with C_h symmetry calculated using the hybrid functional HSE06 with spin-orbit coupling. The excitation from the second-highest occupied level to the lowest unoccupied level of the ground state (denoted as 0-1-1) is considered.

Supplementary References

1 Wang, D. *et al.* Determination of Formation and Ionization Energies of Charged Defects in Two-Dimensional Materials. *Physical Review Letters* **114**, 196801-196801 (2015).

2 Xia, S. *et al.* Evaluation of Charged Defect Energy in Two-Dimensional Semiconductors for Nanoelectronics: The WLZ Extrapolation Method. *Annalen der Physik* **532**, 1900318 (2020).

3 Zhang, C. *et al.* Probing Critical Point Energies of Transition Metal Dichalcogenides: Surprising Indirect Gap of Single Layer WSe_2. *Nano Letters* **15**, 6494-6500 (2015).

4 Schweiger, A. & Jeschke, G. *Principles of Pulse Electron Paramagnetic Resonance* (Oxford University Press on Demand, 2001).

5 Ma, H., Govoni, M. & Galli, G. PyZFS: A Python package for first-principles calculations of zero-field splitting tensors. *Journal of Open Source Software* **5**, 2160 (2020).

6 Seo, H., Ma, H., Govoni, M. & Galli, G. Designing defect-based qubit candidates in wide-gap binary semiconductors for solid-state quantum technologies. *Physical Review Materials* **1**, 075002 (2017).

7 Ivády, V., Simon, T., Maze, J. R., Abrikosov, I. & Gali, A. Pressure and temperature dependence of the zero-field splitting in the ground state of NV centers in diamond: A first-principles study. *Physical Review B* **90**, 235205 (2014).

8 Jelezko, F., Gaebel, T., Popa, I., Gruber, A. & Wrachtrup, J. Observation of coherent oscillations in a single electron spin. *Physical Review Letters* **92**, 076401 (2004).

9 Peter, Y. & Cardona, M. *Fundamentals of Semiconductors: Physics and Materials Properties* (Springer Science & Business Media, 2010).

10 Manson, N. B., Harrison, J. P. & Sellars, M. J. Nitrogen-vacancy center in diamond: Model of the electronic structure and associated dynamics. *Physical Review B* **74**, 104303-104303 (2006).

11 Zhang, Z. *et al.* Defect-nucleated phase transition in atomically-thin WS_2. *2D Materials* **8**, 025017 (2021).

12 Edelberg, D. *et al.* Approaching the intrinsic limit in transition metal diselenides via point defect control. *Nano Letters* **19**, 4371-4379 (2019).

13 Dronskowski, R. & Bloechl, P. E. Crystal orbital Hamilton populations (COHP): energy-resolved visualization of chemical bonding in solids based on density-functional calculations. *Journal of Physical Chemistry* **97**, 8617-8624 (1993).

14 Henkelman, G., Arnaldsson, A. & Jónsson, H. A fast and robust algorithm for Bader decomposition of charge density. *Computational Materials Science* **36**, 354-360 (2006).
